# Salt Marsh as a Coastal Filter for the Oceans: Changes in Function with Experimental Increases in Nitrogen Loading and Sea-Level Rise

**DOI:** 10.1371/journal.pone.0038558

**Published:** 2012-08-07

**Authors:** Joanna L. Nelson, Erika S. Zavaleta

**Affiliations:** Environmental Studies Department, University of California Santa Cruz, Santa Cruz, California, United States of America; MESC; University of South Alabama, United States of America

## Abstract

Coastal salt marshes are among Earth's most productive ecosystems and provide a number of ecosystem services, including interception of watershed-derived nitrogen (N) before it reaches nearshore oceans. Nitrogen pollution and climate change are two dominant drivers of global-change impacts on ecosystems, yet their interacting effects at the land-sea interface are poorly understood. We addressed how sea-level rise and anthropogenic N additions affect the salt marsh ecosystem process of nitrogen uptake using a field-based, manipulative experiment. We crossed simulated sea-level change and ammonium-nitrate (NH_4_NO_3_)-addition treatments in a fully factorial design to examine their potentially interacting effects on emergent marsh plants in a central California estuary. We measured above- and belowground biomass and tissue nutrient concentrations seasonally and found that N-addition had a significant, positive effect on a) aboveground biomass, b) plant tissue N concentrations, c) N stock sequestered in plants, and d) shoot:root ratios in summer. Relative sea-level rise did not significantly affect biomass, with the exception of the most extreme sea-level-rise simulation, in which all plants died by the summer of the second year. Although there was a strong response to N-addition treatments, salt marsh responses varied by season. Our results suggest that in our site at Coyote Marsh, Elkhorn Slough, coastal salt marsh plants serve as a robust N trap and coastal filter; this function is not saturated by high background annual N inputs from upstream agriculture. However, if the marsh is drowned by rising seas, as in our most extreme sea-level rise treatment, marsh plants will no longer provide the ecosystem service of buffering the coastal ocean from eutrophication.

## Introduction

Human activity has altered biotic and abiotic environmental controls at rates, scales, and in combinations that are unprecedented: the hydrologic cycle, biodiversity, land cover, the use of biological productivity, water quality, and the cycling of nitrogen (N) have all changed at global scales [1], [2], [3]. Multiple global environmental changes converge in particular at the land-sea interface, with anthropogenic disturbances originating from both the marine and terrestrial realms, making this an important place to study interactions.

Sea-level rise (due to climate change) and N pollution are two dominant drivers of global change affecting ecosystems; although both are recognized threats to coastal salt marshes, their interacting effects are unknown. Coastal salt marshes are highly productive ecosystems [4], [5] that provide a number of ecosystem services, including interception of watershed-derived nitrogen (N) and other pollutants before they reach the ocean [6], [7], [8]. Salt marsh is a threatened habitat in California, having lost 75 to 90 percent of its historic extent [9], [10].

Sea-level rise is changing the character and location of the land-sea interface and therefore the existence, distribution, and potential migration of salt marshes [11], [12]. Salt marsh existence depends on the relative elevation difference between sea level and the marsh platform, determined not only by sea level, but by marsh subsidence, erosion, and rate of sediment delivery or organic-matter accretion. A recent study projects a global sea-level rise by 2100 of 0.5 to 1.4 meters above the 1990 level [13], which exceeds the 2007 IPCC maximum estimate of 0.6 meters. Sea level affects marsh distribution and density through the mechanisms of waterlogging and salinity stress [14], [15]. Resilience of salt marsh to sea-level rise depends on the ability of a) halophytes to migrate upland, or b) the marsh platform to rise at a similar pace due to sediment accretion or organic-matter accretion. Whether coastal marshes can keep pace with accelerating sea-level rise is an open question [12]. At our study site, Elkhorn Slough, marshes are unlikely to keep pace with sea-level rise because the Slough has physical, hard shoreline barriers – levees, tide gates, and rip-rap – that will likely obstruct marsh migration towards the uplands. Secondly, paleoecological research in Elkhorn Slough indicates the rate of sediment accretion on the marsh platform has been 2–5 mm/yr for the past 50 years, and 1–2 mm/yr in the 200 years before that [16], which is lower than the predicted rate of 5–7 mm/yr of sea-level rise [2]. In this estuary, marsh-platform building is dominated by sediment accretion [16]. Elkhorn Slough marshes have been stable over the past five years, as sedimentation of 3–4 mm/yr has been closely matched by subsidence [17].

**Figure 1:**
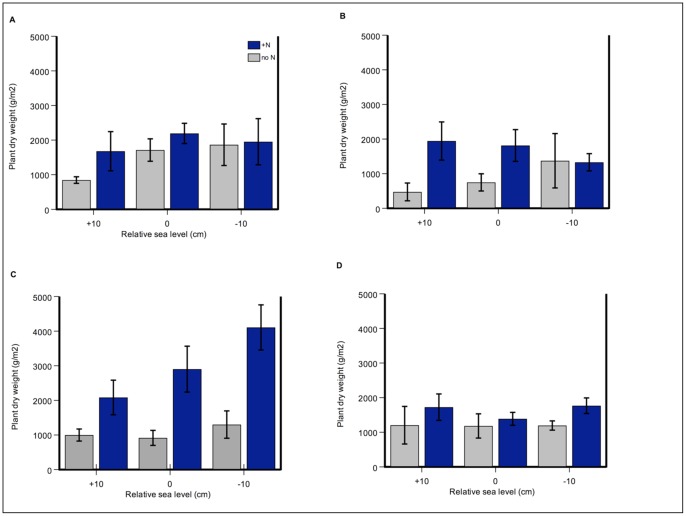
Nitrogen addition increased aboveground marsh biomass. Aboveground salt marsh plant biomass (g m^−2^) in a) July 2008; b) Nov 2008; c) July 2009; and d) Nov 2009 harvests. Salt marsh plant species are the dominant *Sarcocornia pacifica*, as well as *Jaumea carnosa*, *Frankenia salina,* and *Distichlis spicata*. Four out of five harvests are shown: April 2009 was very similar to November of each year. Control treatment (no N) is shown in grey, and N-addition treatment (+N) in blue. Error bars depict standard error of the mean.

**Figure 2:**
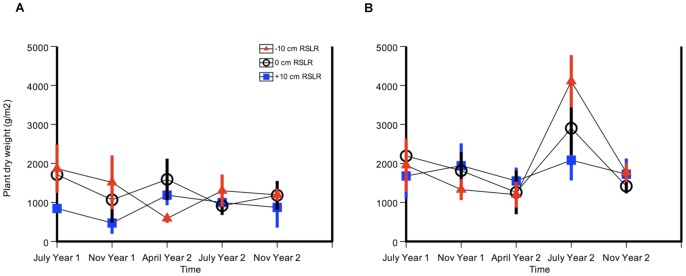
Nitrogen addition increased aboveground marsh biomass most strongly in the second growing season. Aboveground salt marsh plant biomass (g m^−2^) in a time-series depiction of a) control treatment (no N) and b) N-addition treatment (+N). Error bars depict standard error of the mean.

**Table 1:** Results of statistical analyses (General Linear Model).

Model and response variable	Source	df	F	p	
General Linear Model: repeated measures. Data transformed ln(x+1). Excludes 30cm RSLR					
**Aboveground biomass**					
	N level	1	11.08	**0.006**	
	Elevation	2	1.04	0.39	
	N level * Elev	2	0.90	0.43	
	Error	12			
**Aboveground tissue [N]**					
	N level	1	35.81	**<0.001**	
	Elevation	2	0.08	0.92	
	N level * Elev	2	0.47	0.64	
	Error	11			
	Within subjects: Month	4	5.04	0.002	
**N stored succulent pickleweed**					
	N level	1	13.88	**0.003**	
	Elevation	2	2.99	0.09	
	N level * Elev	2	0.58	0.57	
	Error	12			
	Within subjects: Month	4	17.52	**<0.001**	
	Within: Month* N level	4	4.10	**0.006**	
**N stored all species**					
	N level	1	64.48	**<0.0001**	
	Elevation	2	1.26	0.32	
	N level * Elev	2	0.48	0.63	
	Error	12			
	Within subjects: Month	3	26.06	**<0.0001**	
General Linear Model					
**Root biomass** data transformed ln(x)					
July 2009 (Nov 2009)	N level	1	0.55 (3.48)	0.47(0.09)	
	Elevation	2	0.47(0.09)	0.64 (0.92)	
	N level * Elev	2	1.64(0.42)	0.23(0.67)	
	Error	12			
**Shoot:root ratio** data needed no transformation					
July 2009	N level	1	12.32	**0.004**	
	Elevation	2	2.14	0.16	
	N level * Elev	2	0.51	0.61	
	Error	12			
Nov 2009	Block	2	2.239	0.223	
	N level	1	0.226	0.659	
	Elevation	2	0.252	0.789	
	N level * Elev	2	0.334	0.735	

Nitrogen pollution, often due to run-off from agricultural and urban lands, has increased exponentially in recent decades [18] and poses one of the greatest threats to estuarine ecological function [19], [20], [21]. The leading sources of added nitrogen are the application of synthetic fertilizer in agriculture and human population growth rates in coastal areas, with associated runoff [22], [23]. Nitrogen supply in salt marshes affects plant productivity and biomass, and plant physiology, such as resource allocation and tissue N content [24], [25], [26].

Nutrient enrichment of coastal and estuarine systems can lead to altered biogeochemical cycles, disruptive or harmful blooms of phytoplankton and macroalgae, changes in food webs and biodiversity [21], and hypoxic or anoxic ocean regions, also called “dead zones” [27], [28], [29]. In the USA, three quarters of all major estuaries have hypoxic “Dead Zones” [30]. Pathways of nitrogen interception in the coastal environment include plant uptake into tissue, denitrification by microbial communities, and burial in sediments [31], [32]. In the present study, we focus on plant uptake by emergent marsh plants and quantify the ecosystem service of the “coastal filter” (*e.g*., [33], [34]) represented by N sequestered.

In our study, we address the question: 1) How do sea-level rise and anthropogenic nitrogen additions affect the salt marsh ecosystem process of nitrogen uptake? This is the first study we are aware of to investigate the presence and type of interactions between the two stressors in an empirical, controlled experiment in temperate salt marsh. Salt marsh plant zonation has been clearly described, including the observation that increased waterlogging through relative sea-level rise detrimentally affects marsh plant growth and survival [35], [36]. Our novel contribution is to measure the responses of plant growth and N sequestration during simultaneous changes to inundation and N exposure, in order to quantify potential changes to salt marsh ecosystem services. Our objective was to examine changes in the salt marsh's ability to serve as a coastal filter with increases in sea-level rise and nitrogen loading.

In any sea-level-rise scenario, salt marsh plants will experience increased inundation depths and times. We expected the dominant plant, *Sarcocornia pacifica* (Standley) (pickleweed), to decrease in both abundance (biomass) and extent (experimental sea levels where the plants survived) due to ecological drowning. We expected diminished nutrient uptake as plants were physiologically stressed and dying. We anticipated that experimentally raising the marsh platform – analogous to a transplant experiment to higher elevation – (i.e., reducing the frequency of inundation) would improve halophytes' ability to take up nitrogen. Finally, we had a general expectation that nitrogen addition above background levels would increase marsh plant growth, providing antagonistic effects to marsh drowning in the field (e.g., [37], [38]) – but a threshold might exist, where chronic nutrient addition contributed to toxic effects or no longer contributed to growth.

Finally, nitrogen incorporated into plant tissue will continue to cycle when the plant dies or senesces, and decomposes, raising the question of whether plant-bound nutrients have truly been “intercepted” from the ocean. The slower turnover time of nitrogen bound in organic form is generally considered beneficial in buffering the rates and amounts of available-N delivery [24]. How long N is intercepted in standing biomass depends on the lifespan of pickleweed; the plant senesces some succulent tissue annually, but the average lifespan of the perennial plant is unknown. In other N-cycling studies of halophytes – with a focus on temporal dynamics – decomposition is positively influenced by N in tissues, negatively affected by C:N ratios, and occurs in autumn and winter despite lower temperatures [39]; maximum N accumulation in a *Spartina* marsh in Georgia occurred in aboveground tissues in summer and belowground tissues in winter [40]; in a US Northeast *Spartina* marsh, seasonal differences in total N pools of herbaceous species were most strongly influenced by belowground fine root matter and dead macro-organic matter fluxes [41]. The timing of nutrient delivery and plant uptake is important to the efficacy of marsh as a coastal filter [24]. *Sarcocornia* is most productive (with green, succulent, new tissue) in the summer months and dormant (with woody stems) in the winter [37]. Thus, there is a potential “mismatch” in timing in Pacific Coast marshes, where maximum plant production occurs in summer and peak nutrient runoff arrives with winter rains. This timing mismatch could mean that a heightened winter nutrient pulse has relatively greater effect on belowground growth than it does on then-dormant-aboveground marsh plants, as well as that dormant winter plants have a weaker influence on winter nitrogen movement through the marsh and to the coastal ocean. To capture the dynamics of this potential mismatch, we explored marsh response to N addition and sea-level rise simulations by harvesting plants in the months of April, July, and Nov/Dec (spring, summer, and winter).

## Results

### Above- and belowground biomass production

Nitrogen addition increased aboveground marsh biomass (N-level F = 11.08, p = 0.006) (Fig. 1 and 2, Table 1). Nitrogen-addition effects were strongest in Year Two of treatments, particularly in July during the summer growing season (Fig. 1). For example, in July 2009 at −10 cm relative sea level, fertilized plots and unfertilized plots had mean biomass of 4.1 (±0.67) kg m^−2^ and 1.3 (±0.41) kg m^−2^, respectively – a three-fold difference.

In contrast, relative sea-level rise had no significant effect on biomass (RSL F = 1.04, p = 0.39) and did not influence the N response (N-level x RSL F = 0.90, p = 0.43) (Table 1). The only harvest in which both treatments had any type of interactive or synergistic effect was the summer (July) of Year Two, where effects were additive: in the presence of N-addition, biomass decreased linearly with relative sea-level rise (Fig. 1). This pattern differed from the first year of the experiment, where in the absence of N-addition (ambient conditions), biomass decreased linearly with relative sea-level rise in both July and November (Fig. 1).

Within each year, plant growth increased with N-addition most strongly in the summer, and biomass was highest in July of Year Two (Fig. 1 and 2). Pairwise comparisons of the significant N effect on biomass indicated that July of the first year was significantly different than all three of the second-year harvests (factor  =  season, p = 0.04, p<0.001 and p<0.001). In the second year, April biomass was lower than July of that same year (p = 0.04). The significant N effect on biomass was also apparent in a main-effects test (averaged over all elevations and seasons) (t = −4.81, p<0.001) (Table 2).

**Table 2:** Results of statistical analyses (paired t-test).

Main effects of N		df	t	p
Paired t-test of no N and +N treatments				
**Aboveground biomass**		44	−4.81	**<0.0001**
**N stored all species**		44	−6.81	**<0.0001**

**Figure 3:**
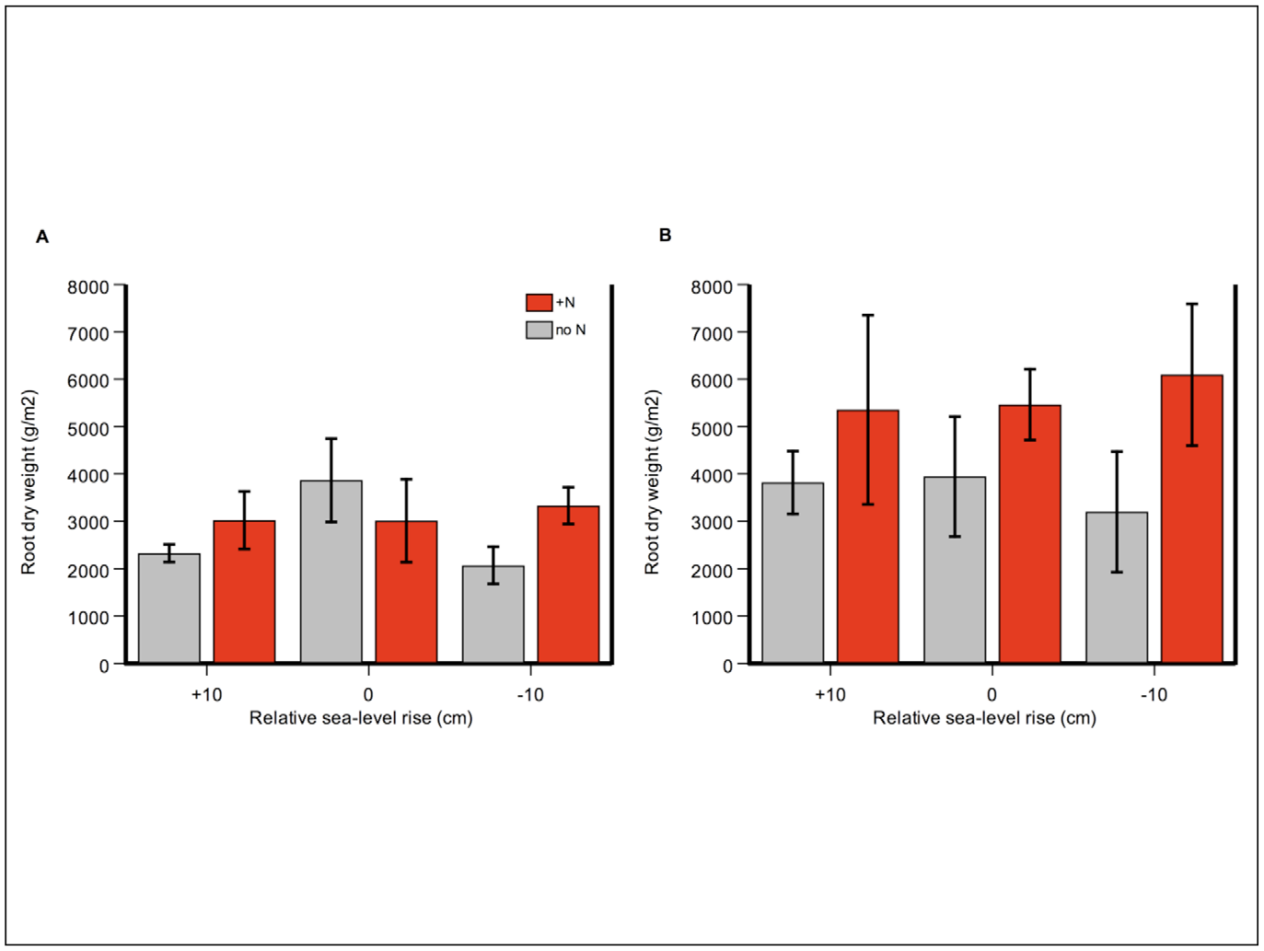
Marsh root biomass was higher in winter than summer. Salt marsh root biomass is almost twice as high in the dormant season of winter as in summer. Root biomass (g m^−2^) in a) July 2009, and b) November 2009. Control treatment (no N) is shown in grey, and N-addition treatment (+N) in red. Error bars depict standard error of the mean.

Root biomass tended to increase with nitrogen addition in November (N level F = 3.48, p = 0.09), but relative sea-level rise did not have a discernible effect (RSL July F = 0.47, p = 0.635; RSL Nov, F = 0.09, p = 0.915) (Fig. 3 and Table 1). Root biomass in November 2009 was almost double that of July 2009: November's fertilized root biomass at −10 cm relative sea level averaged 6096 g (± 1527), compared to 3330 g (± 419) in July, a 183% increase.

**Figure 4:**
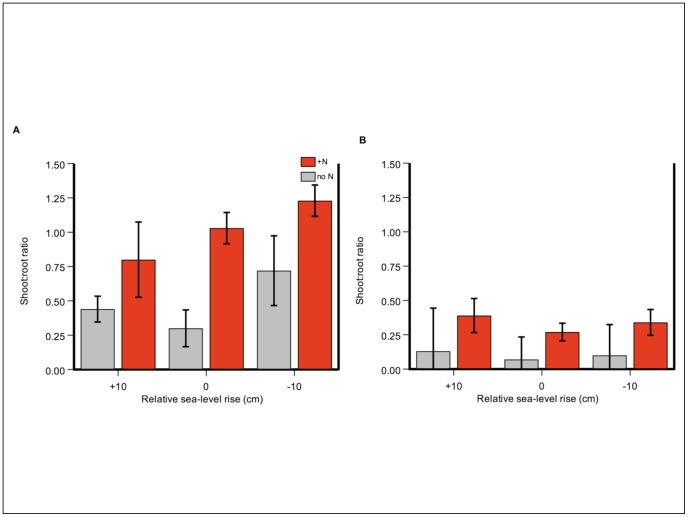
Nitrogen addition strongly increased shoot: Root ratios in the summer growing season, but not the winter. Because of the strong effect of N increasing aboveground biomass in July and modest effect of increasing belowground biomass in November, N strongly increased shoot:root ratios in July. Shoot:root ratios (unitless) in a) July 2009, and b) November 2009. Control treatment (no N) is shown in grey, and N-addition treatment (+N) in green. Error bars depict standard error of the mean.

Because of the strong effect of N increasing aboveground biomass in July and modest effect of increasing belowground biomass in November, N strongly increased shoot:root ratios in July (N level F = 12.31, p = 0.004) (Fig. 4). Changing relative sea level did not exert a significant effect on shoot:root ratios (RSL F = 2.14, p = 0.16) or influence the N treatment (N-level x RSL F = 0.51, p = 0.61).

There was very little evidence for spatial variation in marsh growth, in that a test for a block effect was non-significant in all analyses. Although there was a strong and interpretable overall response to treatments, salt marsh responses varied temporally, by season.

#### Extreme sea-level rise treatment

In the highest simulated sea-level rise of 30 cm, all salt marsh plants died in Year Two of the experiment, between spring and summer. N-addition led to greater biomass in only one of three harvests with living plants, in winter of the first year (Fig. S1).

### Plant tissue nitrogen

#### Nitrogen concentration

Nitrogen concentration (mg N g^−1^) in aboveground plant tissue increased strongly in plots with N-addition (N-level F = 35.81; p<0.001) (Fig. S2 and Table 1). Similar to results for biomass, simulated sea-level rise did not have an effect (RSL F = 0.08, p = 0.92), and there was no interaction between the treatments (F = 0.47, p = 0.64) (Table 1). There were significant within-subject (within-plot) effects of season (F = 5.04, p = 0.002), leading to an exploration of temporal variation: N concentration in July of the first year was significantly different than N concentrations in July and November of the second year (factor = season, p<0.001 for each comparison), and N concentration in April of the second year was significantly different than July or November of the same year (factor = season p<0.001 and p = 0.002).

Treatment effects on plant N concentration were most apparent in Year Two, as with biomass, but in the dormant season of November rather than the growing season of July. At a maximum – November 2009 in the +10 cm sea-level rise plots –pickleweed (*S. pacifica*) succulent tissue had a concentration of 37.17 (±23.9) mg N g^−1^plant tissue when fertilized compared to 9.06 (±0.27) mg N g^−1^ plant tissue in controls, a 410% difference (Fig. S2).

N addition significantly increased root-N concentration in coarse roots only, in November (N-level F = 25.32, p<0.001; RSL F = 1.50, p = 0.26). There were no discernible treatment effects on fine roots (N-level F = 0.002, p = 0.96; RSL F = 0.50, p = 0.62).

#### Extreme sea-level rise

In the highest simulated sea-level rise of 30 cm, N concentration in aboveground tissues increased significantly with added inorganic N only in April of the second year (N-level F = 20.41, p = 0.01) (Fig. S1).

#### Plant nitrogen sequestration

**Figure 5:**
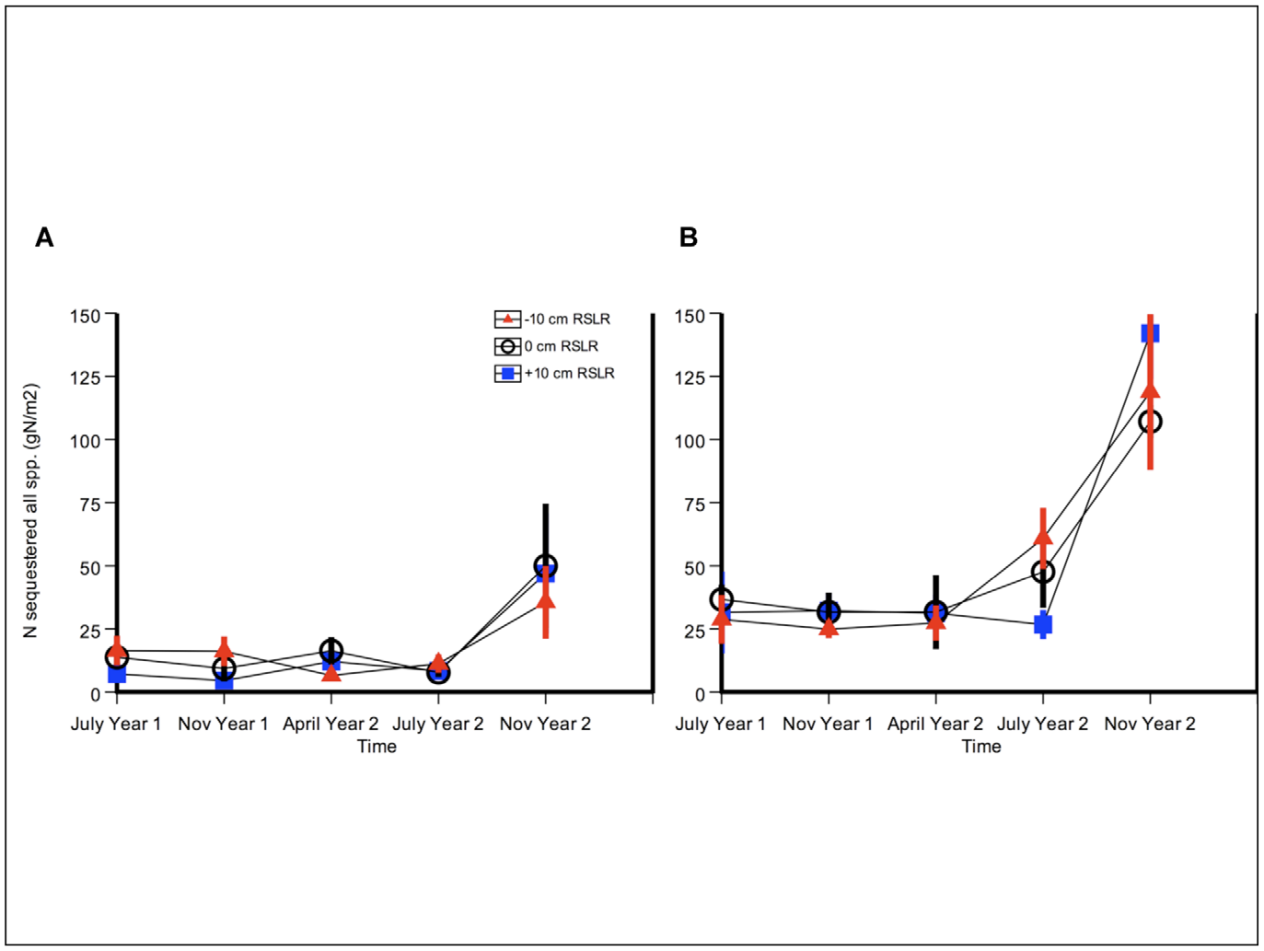
Salt marsh functions as a coastal filter, with a winter maximum across all halophyte species. Nitrogen sequestered (gN m^−2^) in a time-series depiction of a) control treatment (no N) and b) N-addition treatment (+N). Error bars depict standard error of the mean.

**Figure 6:**
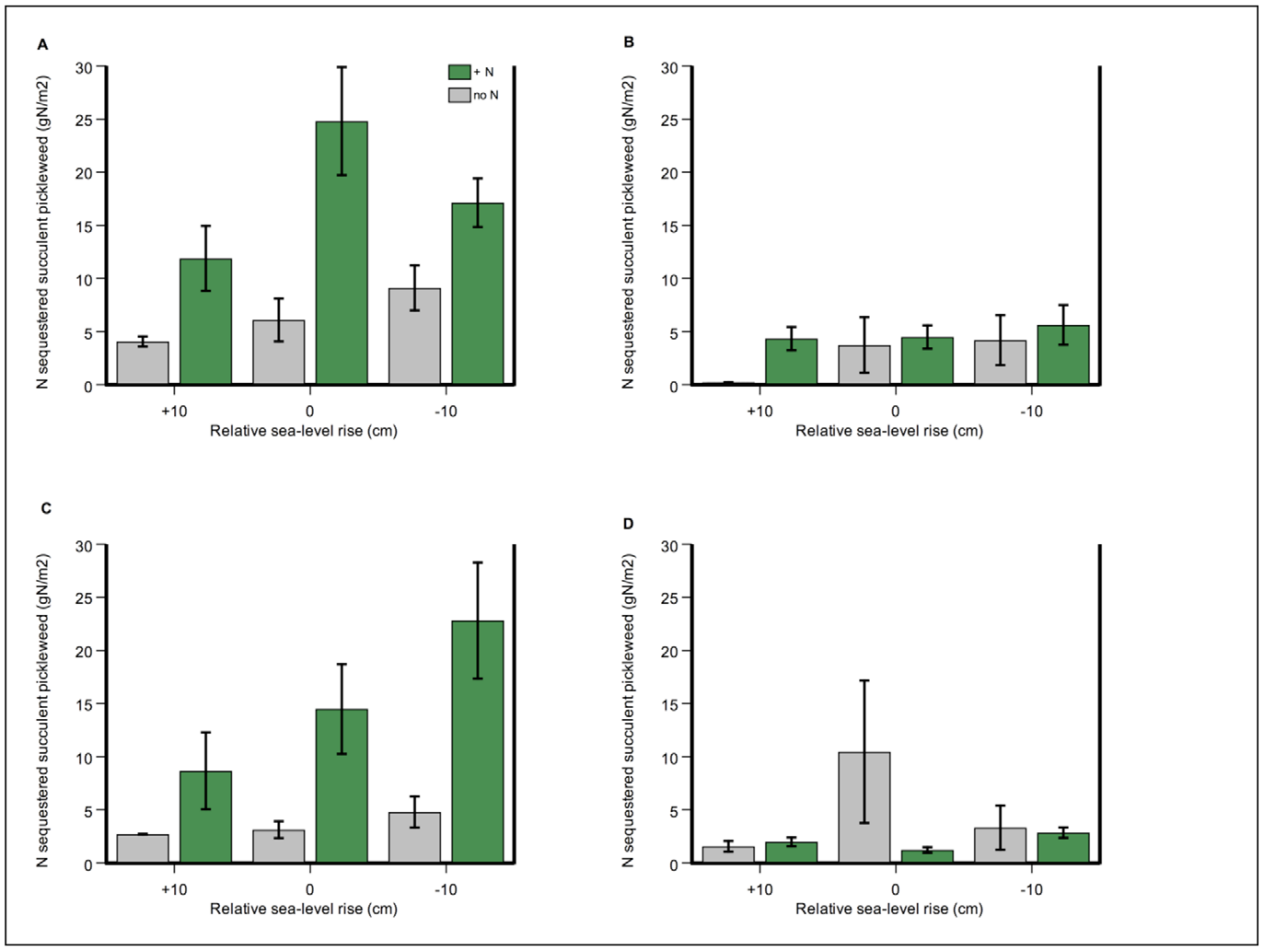
Salt marsh functions as a coastal filter in new-growth, succulent pickleweed, especially in summers. At a maximum, plants with added inorganic nitrogen sequestered more than four times as much as N as controls. Total N sequestered in *S. pacifica* new-growth tissue (gN m^−2^) in a) July 2008; b) November 2008; c) July 2009; d) November 2009. Four out of five harvests are shown: April 2009 was very similar to November of each year. Error bars depict standard error of the mean.

**Figure 7:**
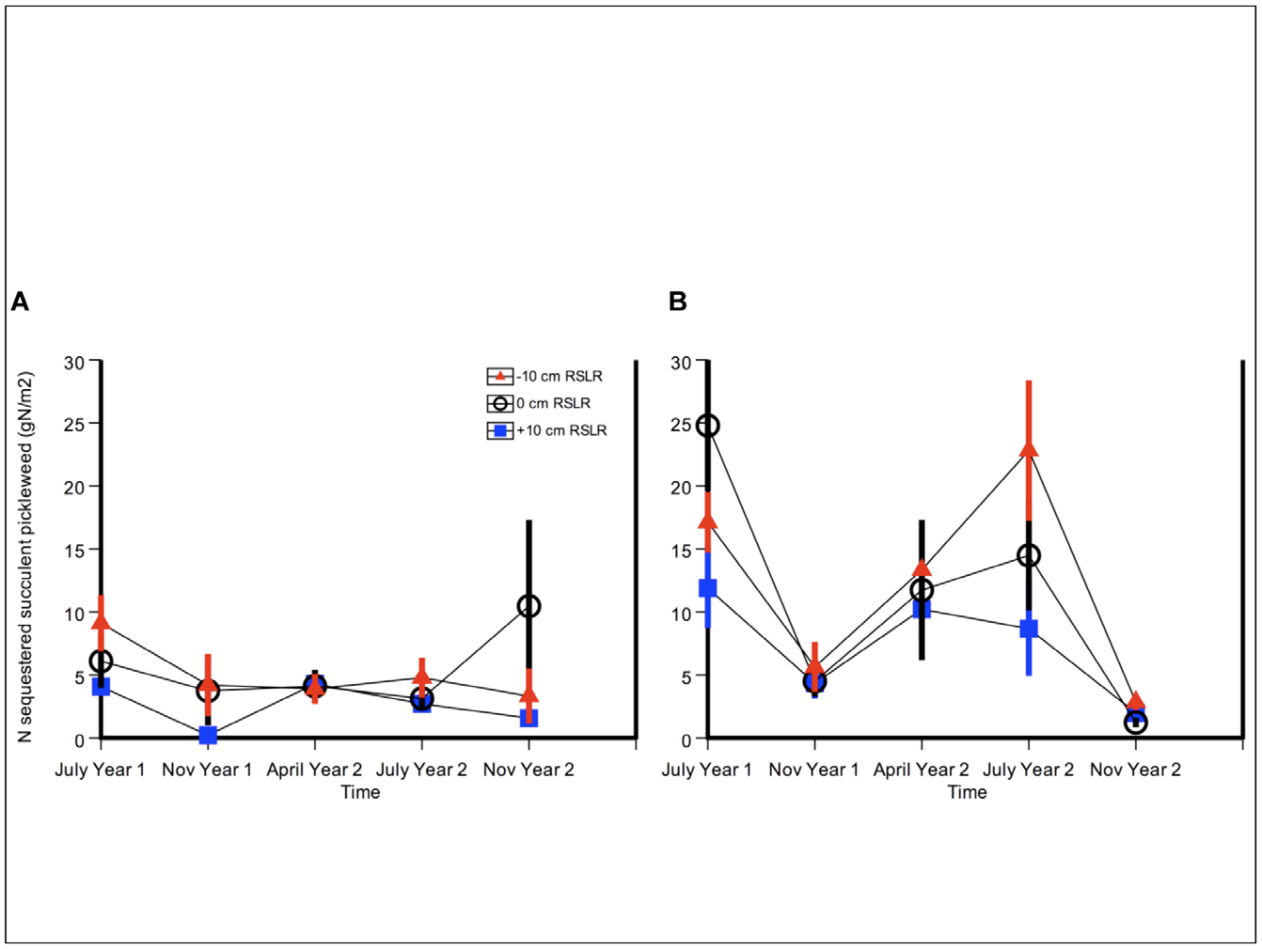
N sequestered in succulent pickleweed peaked in summer in added-N treatment. Total N sequestered in *S. pacifica* succulent tissue (gN m^−2^) in a time-series depiction of a) control treatment (no N) and b) N-addition treatment (+N). Error bars depict standard error of the mean.

Total nitrogen sequestered in all halophyte species and tissue types – a product of nitrogen concentration and biomass of all species – increased strongly in response to N addition (N level F = 64.48, p<0.0001) (Fig. 5 and Table 1). N sequestered in succulent pickleweed (gN m^−2^) only increased strongly in response to N addition (N level F = 13.88, p = 0.003) (Fig. 6 and 7). Relative sea level did not have a significant effect (RSL F = 2.99, p = 0.09), with no interaction between treatments (N-level x RSL F = 0.581, p = 0.57). Pickleweed sequestered more N in the summer seasons (season (F = 17.53, p<0.001) (Fig. 6), although a significant interaction between season and N-level makes this difficult to interpret (season-by-N level F = 4.10, p = 0.006). At a maximum, fertilized plants stored more than four times as much nitrogen as controls: in July 2009 at −10 cm relative sea-level rise, plants sequestered 22.8 (±5.6) gN m^−2^ compared to no-N plots with 4.8 (±1.6) gN m^−2^, a difference of 475 percent (Fig. 6 and 7). At that same time and plot elevation, biomass increased at a lower rate of 316 percent (4107 g m^−2^ average fertilized biomass vs. 1300 g m^−2^ average unfertilized biomass) (Fig. 1).

#### Extreme sea-level rise treatment

There was no significant effect of N addition on N stored in plots with 30 cm of simulated sea-level rise (F = 0.69, p = 0.45). However, there was a within-plot effect of season (F = 4.48, p = 0.05) (Fig. S1).

## Discussion

Important ecosystem functions and services provided by temperate salt marsh are at risk of being diminished by directional, ecological change. Multiple global changes can interact to dampen, amplify, or add to each other's effects, adding complexity that is important to address in our understanding of ecological processes. In this study, the global changes of sea-level rise and nitrogen pollution have strong effects on salt marsh productivity and nutrient cycling. First, salt marshes buffer terrestrial N pollution through plant uptake; however, sea-level rise quickly diminishes salt marsh extent. Second, halophytes sequester more nitrogen – through growth and increased tissue N concentrations – with inorganic N addition. Third, there are seasonal variations in response to treatments – where plants grow more, sequester more nitrogen in succulent tissue, and generally respond more strongly to the combination of treatments in the summer growing season rather than the winter dormant season – with the exception of N sequestration across all species, which was highest in November of the second year of the study.

This is the first study to examine the interaction of nitrogen pollution and sea-level rise on the capacity of temperate salt marshes to intercept land-derived N to protect ocean functioning. Our results suggest the plants serve as a robust N trap, or coastal filter. Additionally, in the case of Coyote Marsh, Elkhorn Slough, this function is not saturated. However, if the marsh is drowned by rising seas – as it was in the most extreme sea-level rise simulation – the plants will no longer provide the ecosystem service of buffering the ocean from eutrophication.

### Interacting effects of N addition and sea-level rise on salt marsh

In our investigation of salt marsh as a coastal filter, simulated sea-level rise reduced marsh resilience to N loading, and additions of inorganic N led to more N uptake into plants. Nitrogen had the dominant effect on plant growth and N sequestration; simulated sea-level rise only had a significant effect on plant growth and N uptake when waterlogging killed plants; and in the peak growing season of the second year (summer), the effects of the two perturbations were additive: in the presence of N-addition, plant biomass and N sequestration decreased linearly with relative sea-level rise.

Marshes buffer estuarine waters from N loading through plant uptake. In response to N addition, N concentrations increased in succulent, annual tissue of the dominant marsh plant, pickleweed; growth and shoot:root ratios of all four marsh species increased, with a larger proportion of N-rich shoots relative to lower-N roots. Together, these three factors drove the magnitude of N sequestered on a per area basis, which was four times higher in fertilized plots.

It is notable that in an estuarine environment with *high* concentrations of nitrate in the main channel water (up to 250–300 µM NO_3_-N in winter [42], [43]), salt marsh plants continued to be N-limited (indicated by increased biomass with N addition). High nitrate concentrations in the main channel of the Slough fuel productivity that can be categorized as beyond “eutrophic” to “hypertrophic” [44]. Therefore, although “control” plots are bathed in high concentrations of nitrogen during tidal inundation, “+N” treatment plots show still higher growth and continued uptake.

Salt marsh plants in Coyote Marsh were vulnerable to the extreme sea-level rise simulation, in that all plants died by the middle of the second year of the experiment and ceased to provide the filtering function of N uptake. This result is consistent with the estuarine literature [35], [36], [45]; however, our novel contribution was to look at N sequestration as plants were inundated, physiologically stressed, and dying. In the simulated sea-level rise of +30 cm, plants did not have significantly higher N concentrations in their tissue than other elevation treatments, so there was no compensatory effect of more N sequestration per biomass. Therefore, a decrease in biomass indicates a proportional decrease in N uptake, implying that sea-level rise will severely diminish the buffering function of salt marsh.

Plants exposed to the less extreme simulation of sea-level rise (+10 cm) survived throughout the two-year experiment, and their biomass did not differ significantly from that of the ambient marsh platform. These results suggest plant growth was not adversely affected by the 10-cm sea-level rise treatment, and that – unsurprisingly – the rate and magnitude of tidal inundation matter in terms of species' responses and survival. However, plant biomass reached a one-time maximum in plots with the higher-elevation treatment (summer of the second year), supporting the idea that a marsh platform at a higher elevation in the range of MHW to MHHW could promote marsh plant growth. Although the simulated sea-level rises we imposed were sudden, rather than the gradual rate predicted (5–7 mm/year eustatic rise in 50 years [2]), the total amount of rise is on par with IPCC 2007 predictions (25–35 cm in the next 50 years).

In contrast to aboveground measures, root biomass did not respond as strongly to N-addition in our two-year study, either showing no response or tending to increase. There is disagreement in the literature about the vulnerability of salt marshes to eutrophication, which centers on belowground responses. Some results indicate that nutrient-enriched sediments, such as treated sewage sediments, have no growth-retarding effects on marsh plants [46], while other studies show relatively lower root growth in marshes with nutrient addition [47], [48] which can contribute to subsidence of the marsh platform [48], [49]. Halophyte roots' potential contribution to building marsh platform elevation, and therefore marsh sustainability, points to the importance of measuring both above- and belowground biomass, and we observed a much stronger growth response to N addition in aboveground biomass.

### Salt marsh capacity to act as a coastal filter

As compared to other studies of salt marsh interception of land-derived N, Elkhorn Slough salt marsh appears to serve as a robust N trap. Notably high interception of externally added N has also been shown in the Great Sippewissett Salt Marsh, New England [6], even after 30 years of experimental fertilization treatments at low, high, and extra-high fertilization rates (0.9, 2.6 and 7.8 g N m−2 wk−1, respectively, in a N-P-K mix). However, in another New England study, salt marsh vegetation exposed to ∼70 µM NO_3_^−^ reached a saturation point for uptake, and became less effective at pollution control than the reference systems [50]. In a study in Portugal, the capacity of salt marshes to retain N depended on the age of the marsh, where the oldest marshes retained the most [51]. All of the above studies focused on low-marsh *Spartina spp*., cordgrass, whereas there is no *Spartina* in Elkhorn Slough; species differences need to be taken into account. In terms of N application rates and loads, our study is closely matched to the extra-high fertilization treatment in Great Sippewissett Salt Marsh and exceeds the ∼70 µM NO_3_^−^ treatment by four orders of magnitude. Other studies of nutrient enrichment in U.S. Pacific Coast, pickleweed-dominated salt marsh have shown that urea addition increases salt marsh productivity, alters community structure [37], [52] and increases susceptibility to species invasions [53]. Organic forms of N, such as urea, depend on microbial mineralization for plant availability and are therefore considered “slow-release” applications [54]. In a greenhouse study of Elkhorn Slough pickleweed, a toxicity threshold was reached upon adding >7.0 g l^−1^ of urea-N [55], resulting in plant death. In comparison, our +N treatment exceeded that threshold amount – we applied 15 g l^−1^ of ammonium-nitrate-N biweekly – and was delivered in plant-available form (inorganic N), implying that “nitrogen burn” or lethal toxicity effects could have appeared more quickly than a slow-release form, yet did not over the course of two years. The difference in the greenhouse- and field study reinforces the importance of experiments that simulate real ecosystem conditions as closely as possible: plants in the field could intercept higher N loads than greenhouse cuttings. The difficulty in comparing Elkhorn Slough uptake to other marshes is that studies of interception of land-derived N were done primarily in Northeast U.S. *Spartina* marshes, and studies of Pacific Coast U.S. pickleweed marshes largely measured productivity or community structure. Taking into account species differences, organic or inorganic N application, and a range of response variables, Elkhorn Slough marsh appears to intercept a notably high amount of nitrogen.

### Seasonality

The peak marsh growing season is summer in Elkhorn Slough marshes, but the highest concentrations of nutrients are delivered with winter rains (November – March). U.S. Pacific Coast marshes, in a Mediterranean-type climate, differ in this potential mismatch in timing from other North American marshes. For example, in the Mississippi River Delta, spring floods deliver N synchronous with peak growth of wetland grasses [56], [57], [58] (note, however, that N entering the Mississippi River Delta is the highest load in the US by an order of magnitude [30], so the synchronicity of delivery and plant uptake does not imply complete uptake.) Marsh plant uptake helps buffer nitrogen loading, as do terrestrial vegetative buffer strips in Elkhorn Slough (e.g., [59]), but they do not seem to be a comprehensive N trap given the timing mismatch [68]. Marsh plant uptake of N is not, therefore, a substitute for policies that reduce fertilizer N inputs and losses from land [22], [60], [61].

In the summer growing season, marsh plants grew more with N addition, relative to controls, than any other season. Nitrogen sequestration – our measure of the coastal filter – in succulent pickleweed (new-growth only), reached a maximum in the second summer. On the other hand, interestingly, our two-year experiment shows that a) marsh plants are taking up excess N in each season studied; and b) the total nitrogen sequestered, in all plant species and tissue types, reached a maximum in November of the second year, which demonstrates closer alignment in the timing of uptake and delivery of nitrogen in the estuary.

### Nitrogen interactions with other global changes

Other studies exploring multiple-stressor interactions have shown varied responses to N-loading: dampened or amplified interactions, a switch in source/sink dynamics, and additive effects. In our experiment, when we found formal interactions (synergistic or additive), we found simple, additive interactions between the two changes of simulated sea-level rise and added nitrogen in the first year's summer growing season. In a Chesapeake Bay marsh, N additions led to a plant community shift towards C4 plants that diminished CO_2_ uptake [62]. In a US Northeast *Spartina patens* marsh (Plum Island Sound, MA), short-term N additions created a source of the greenhouse gas nitrous oxide rather than a sink [63]; the role of salt marsh as a sink showed temporal variation, as did our study, relative to the growing season. In an earlier, ecosystem-scale study in the same estuary, (Plum Island Sound) water-column nitrate additions and predatory-fish reduction created synergistic amplified effects, increasing benthic microalgae biomass significantly in salt marsh creeks [64]. In a serpentine grassland in central California, four simulated, global changes – N deposition, elevated CO_2_ concentration, warming and precipitation – did not interact synergistically in their effects on plant biodiversity; the treatments produced simple, additive combinations of single-factor effects [65]. These studies underscore the importance of assessing potential interactions between multiple human disturbances rather than extrapolating from single-factor experiments, in order to maintain ecosystem functions and services under global change.

### Conclusions and implications for management

Our results have implications for management of both the elevation of marshes and agricultural best-management practices to limit nitrogen losses from land. For example, raising the level of the marsh platform – with halophyte species that play a role as ecosystem engineers [66] or with added dredge sediments [46], [67], [68] – can increase vegetation productivity. Dredge sediment addition has raised questions about nutrient, metal, and pollution concentrations in those sediments. Given sources of sediment that have acceptably low levels of pollutants, sediment addition is an intervention that seems to support marsh survival and sustainability under conditions of relative sea-level rise [67], [68], [69]. In Elkhorn Slough, a potential management action is the adding of dredge materials to bare mudflat, lower in the estuarine intertidal than marsh, to prompt the growth of marsh vegetation [67]; restoration literature suggests that the dominant plant, pickleweed, can recruit from surrounding areas without re-planting efforts [70]. Our results suggest optimal heights for the marsh platform, within the MHW to MHHW range, to promote marsh productivity and N uptake.

Salt marsh distribution will change with sea-level rise – coastal wetlands could establish in areas where they may not have been documented currently and disappear from protected areas [71] – making tools for flexible land-use and conservation of greater importance. Exploring regulation and management strategies to mitigate greenhouse gas emissions and abate nutrient enrichment, at the same time, will be valuable to both conservation of coastal marshes and improvement of ocean water quality [60], [61], [72], [73], [74].

## Materials and Methods

### Study site

**Figure 8:**
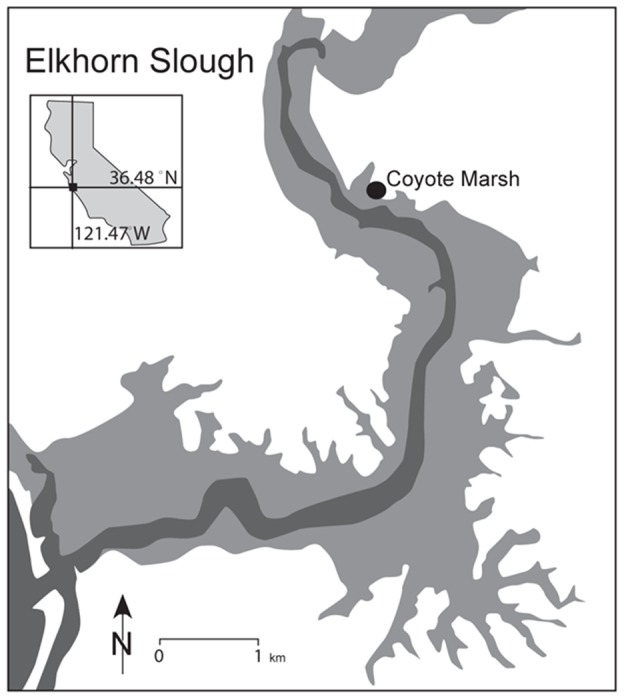
Location of Elkhorn Slough and experiment site. Moss Landing, California, on the coast of Monterey Bay. The experiment site, Coyote Marsh, is located in the Elkhorn Slough National Estuarine Research Reserve.

Elkhorn Slough (36°48′ N, 121°47′ W), located on the central coast of Monterey Bay, California, has one of the largest tracts of coastal salt marsh habitat in California, with 1,147 ha of marsh [75] (Fig. 8). The main channel of the Slough is part of the Monterey Bay National Marine Sanctuary and is surrounded by agricultural lands, with 24% of the slough watershed under production [76], primarily in heavily fertilized strawberries and vegetable row crops.

We established our experiment at Coyote Marsh, a high marsh in the Elkhorn Slough National Estuarine Research Reserve (ESNERR) (Fig. 8). Plant species at the site included *Sarcocornia pacifica* (pickleweed), which was the predominant cover, as well as *Jaumea carnosa* (fleshy jaumea), *Frankenia salina* (alkali heath), and *Distichlis spicata* (salt grass). All necessary permits were obtained for our field study through ESNERR, administered jointly by the National Oceanic and Atmospheric Administration and the California Department of Fish and Game.

#### Nitrogen delivery to the site

Nitrogen is delivered to marshes in surface runoff, groundwater, and inundation with estuarine-ocean water on flood tides. Therefore, although Coyote Marsh is subjected to flood tides no more than 4–6 times a month on average in winter, the marsh plants do get the 250–300 µM NO_3_-N as an ambient dose. In Elkhorn Slough, 66% of nitrate in the main channel comes from terrestrial sources [77], as distinct from ocean upwelling, so even the flood tides are a majority of “land-derived” N.

#### Hydrodynamics and sediment characterization of the site

Elkhorn Slough has only one small, ephemeral river input (Carneros Creek) at the head. After major hydrologic changes to the Slough, it is considered starved of sediment delivery [48]. Finally, Elkhorn Slough is an ebb-dominated estuary [78], which tends to emphasize sediment loss with higher-velocity ebb waters. Paleoecological research indicates that a) the sediments are primarily inorganic (75% inorganic by weight); and b) sediment accumulation rate has been 2–5 mm/yr in the last 50 calendar years, and 1–2 mm/yr in the time period 200–50 years before the present [16]. Sediment size class is categorized as “fine silt”: specifically, particle size distributions show a bimodal peak at 4 and 16 µm [16], [17].

### Experimental design

**Figure 9:**
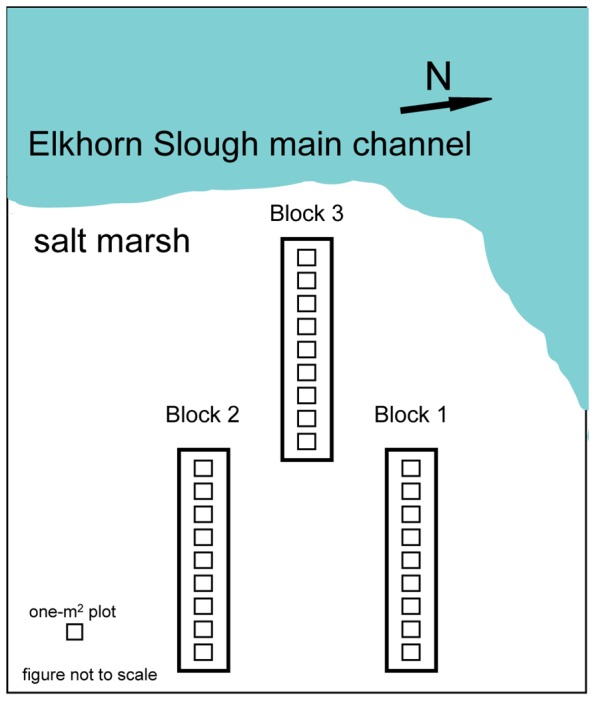
Diagram of block arrangement and experimental design. Coyote Marsh, Elkhorn Slough National Estuarine Research Reserve. Each block contained all treatment combinations (4 elevations and 2 N levels) plus one dig-control plot, for a total of 9 plots per block.

We crossed relative sea-level and nitrogen treatments in a fully factorial design to examine their potentially interacting effects on plant biomass and tissue nutrient concentrations. We used marsh elevation as a proxy for sea-level rise, and chose three elevations – with a fourth extreme sea-level rise simulation. Simulated sea levels were chosen to fall within the spectrum of IPCC (2001) scenarios (where +30 cm was the maximum predicted), or an ecologically significant amount of sedimentation addition [66]. Sediment addition incorporates predictions of more variability in precipitation and storm events with climate change [79]. The simulated sea levels were +10 cm; 0 cm (the ambient marsh platform); and −10 cm, simulating an increase in elevation of 10 cm, which might occur via a) sediment additions from more extreme storms; or b) management interventions to raise the marsh platform to promote marsh-plant survival. The fourth simulated sea level, which we refer to as “extreme sea level treatment” was +30 cm. Nitrogen additions simulated increased N in terrestrial surface runoff. The two levels of N treatment were 300 g N m^−2^ yr^−1^, in the form of ammonium nitrate (NH_4_NO_3_), or no added nitrogen under ambient conditions. The added N is equal to an average five- to ten-fold addition of the conventional fertilizer used in the region on strawberry or vegetable fields [80]. We chose this level in accordance with other studies of marsh interception of land-derived N (e.g., a range of 10–90 times the recommended fertilizer for commercial oat crops [6], [81]) [64], the potential for increased land conversion to agriculture in the watershed [75], and recent and forecasted exponential increases in agricultural synthetic fertilizer use [82], [83]. We accounted for spatial variability across our site by establishing three blocks, each containing all treatment combinations (4 elevations and 2 N levels), for a total of 24 one-m^2^ plots (Fig. 9). Control plots evaluated a possible digging effect by digging up and then replacing otherwise-unmanipulated marsh vegetation. There was one control plot in each block (Fig. 9): having determined that there was no significant digging effect for each analysis, we did not incorporate data from those plots.

### Field methods

#### Elevation

We created the artificial sea-level rise treatment (adapted from [66]) by selecting a 1x1-m plot of marsh, removing vegetation with intact roots in a block of sediment, removing sediment beneath the vegetation layer (either 30 cm or 10 cm depth of sediment), and replacing the vegetation layer. A difference in marsh-plain elevation of 10 cm has been shown to have ecological effects [66], [84]. The side-walls of plots were held in place with hardware cloth and landscape staples. Lowered plots did not have any drainage channels or other simulations of an ebb tide. Similarly, in raised plots, we removed vegetation with intact roots in sediment, in this case adding 10 cm of sediment underneath the root zone. Additional sediment was taken from the sediment-removal treatments in the same marsh: adding sediment beneath the root zone minimized any nutrient subsidies, corroborated by our findings that elevated plots (no N) show no difference in growth from the ambient-marsh-platform plots. The sediment did not compact any more than one cm (all plots were ≥9 cm elevated at the end of two years). The sediment in the raised plots could drain more readily out the sides of the hardware-cloth retaining structures than lowered plots, which could release marsh plants from waterlogging and salinity stress [18]. Each plot was at least 3 m away from any other plot.

#### Extreme sea-level rise simulation

Marsh plots lowered 30 cm simulated a sea-level rise that we estimate to be greater in magnitude than 30 cm, because the plots did not drain and had no system to simulate an ebb tide. Plants were inundated in water with a salinity of ∼35 (practical salinity units), typical of the main channel Slough and the Pacific Ocean. Plots did drain occasionally, with no intervention, in a pattern that was not correlated with any variables we measured. We refer to this scenario as “extreme sea-level rise simulation” since it is a rapid and almost-continual inundation of marsh.

#### Nutrient addition

We added ammonium nitrate (NH_4_NO_3_) to designated plots in the amount of 15 gN m^-2^ every two weeks. We did not fertilize during July and August of each year, because summer nutrient levels in the Slough are lowest and fertilizer applications are low, becoming high again in October [43]. Therefore, we added a total of 300 gN m^−2^ yr^−1^ to fertilized plots. We dissolved NH_4_NO_3_ pellets in 1 L of main-channel Slough water and added them to treatment plots; we added 1 L of Slough water to each control plot.

#### Biomass harvest

We measured the impacts of sea-level change and nitrogen addition on plant biomass, above- and belowground, and plant physiological measures of tissue nitrogen concentration and resource allocation. We harvested a 10×50 cm swath of aboveground vegetation from each meter-square plot on the following dates: July and November 2008, and April, July and November 2009. The swath was taken from a randomly-chosen quarter of a plot with the following constraints: the 50-cm edge was always internal to the plot to avoid edge effects, we harvested a given 10×50 cm area only once in the two years, and we stopped harvesting when all plants in a plot were visibly dead. Once harvested, we sorted plants by species. We separated succulent (new) and woody (perennial) tissue for *Sarcocornia pacifica* only. All plant material was dried in a laboratory oven at 60°C for at least 48 hours; weighed; and a portion ground with a ball mill (Spex 8000, Spectrum Chemicals and Laboratory Products, CA and NJ, USA). We used a C:N analyzer (Elementar varioMAX, Elementar, Germany) in order to obtain tissue nitrogen concentration.

In the second year only (2009), we harvested root biomass with a 5-cm-diameter sediment corer, taking 20-cm-deep cores. We isolated plant material through root-washing by hand and categorized roots as fine or coarse. The approximate diameter cutoff between fine and coarse roots was 0.5 mm. We dried the roots in a laboratory oven at 60°C for at least 48 hours, weighed them, and ground all material in a ball mill. We analyzed %N in November root data only (due to limited sample size, C:N analyzed with a Costech ECS 4010, Costech, CA, USA).

### Analytical methods

To assess treatment effects on plant aboveground biomass, we grouped all plant species (which includes succulent and woody tissue biomass of *Sarcocornia pacifica*) in each plot and used a General Linear Model with repeated measures in SYSTAT v12 (Systat Software. Inc., Chicago, IL, USA). We tested for a block effect, and where it was insignificant – in all analyses but one – removed it as a factor. Therefore, independent factors were N-level, relative sea-level (RSL) and their interaction. We log-transformed biomass data to conform to a normal distribution. The repeated measures analysis incorporated all 24 plots over 5 harvests (July, November, April, July November). Similarly, to assess treatment effects on plant tissue nitrogen concentration (mg N per gram of plant tissue), we used a General Linear Model with repeated measures analysis, where data were log-transformed. We ran a post-hoc comparison for repeated measures, with a Bonferroni correction for pairwise comparisons, to assess which seasons might be different than each other. To assess experimental effects on root biomass and shoot:root ratios, we used a factorial ANOVA on each of two harvests. In any analysis where there were significant interactions, we explored the data visually to interpret patterns. To test for main effects of one factor, N addition, we used a paired t-test between the groups: average of response variable at reference-level of N (no addition) and average of response variable with added N. We set the significance level for all analyses at α  = 0.05, *a priori.*

## Supporting Information

Figure S1 **Marsh plants are vulnerable to sea-level rise simulation.** Simulation of +30 cm sea-level rise resulted in the death of all salt marsh plants before the summer of Year Two of the experiment (bar graph), where plant tissue N concentrations increased with N treatment (XY graph). Error bars depict standard error.
(TIF)

Figure S2 **Nitrogen concentration in aboveground plant tissue increased strongly in plots with nitrogen addition.** N concentration ([N]) (mgN g^−1^ plant tissue) in a) July 2008; b) Nov 2008; c) July 2009; and d) Nov 2009 harvests. Four out of five harvests are shown. Control treatment (no N) is shown in grey, and N-addition treatment (+N) in green. Error bars depict standard error of the mean.
(TIF)
