# Supplementary figures and images for: Salt Marsh as a Coastal Filter for the Oceans: Changes in Function with Experimental Increases in Nitrogen Loading and Sea-Level Rise
Source: PLoS One. 2012 Aug 7;7(8):e38558. doi: 10.1371/journal.pone.0038558 (PMC3413704; doi:10.1371/journal.pone.0038558)

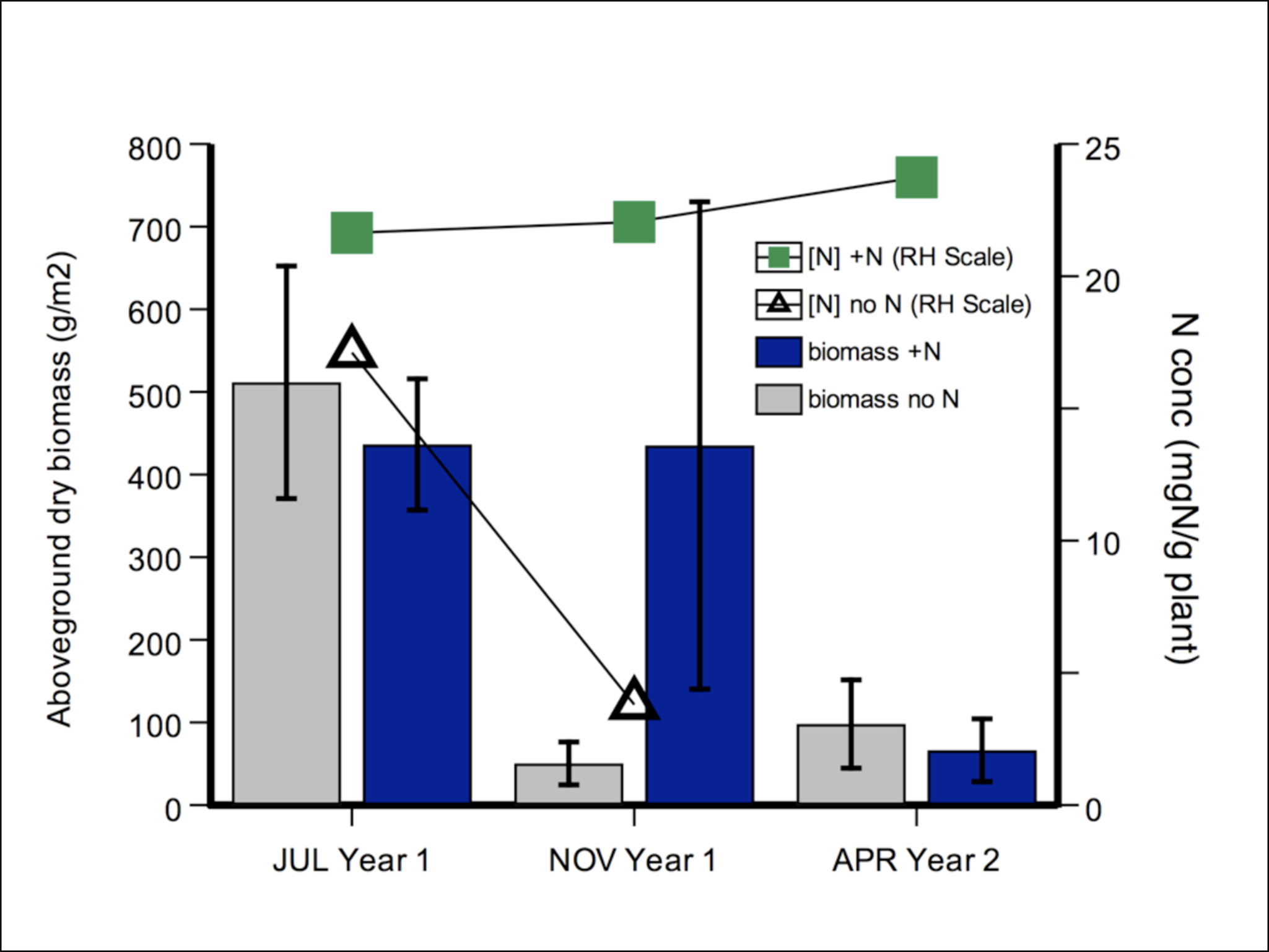

Supplement: Figure S1 — Marsh plants are vulnerable to sea-level rise simulation. Simulation of +30 cm sea-level rise resulted in the death of all salt marsh plants before the summer of Year Two of the experiment (bar graph), where plant tissue N concentrations increased with N treatment (XY graph). Error bars depict standard error. (TIF) [file pone.0038558.s001.tif]

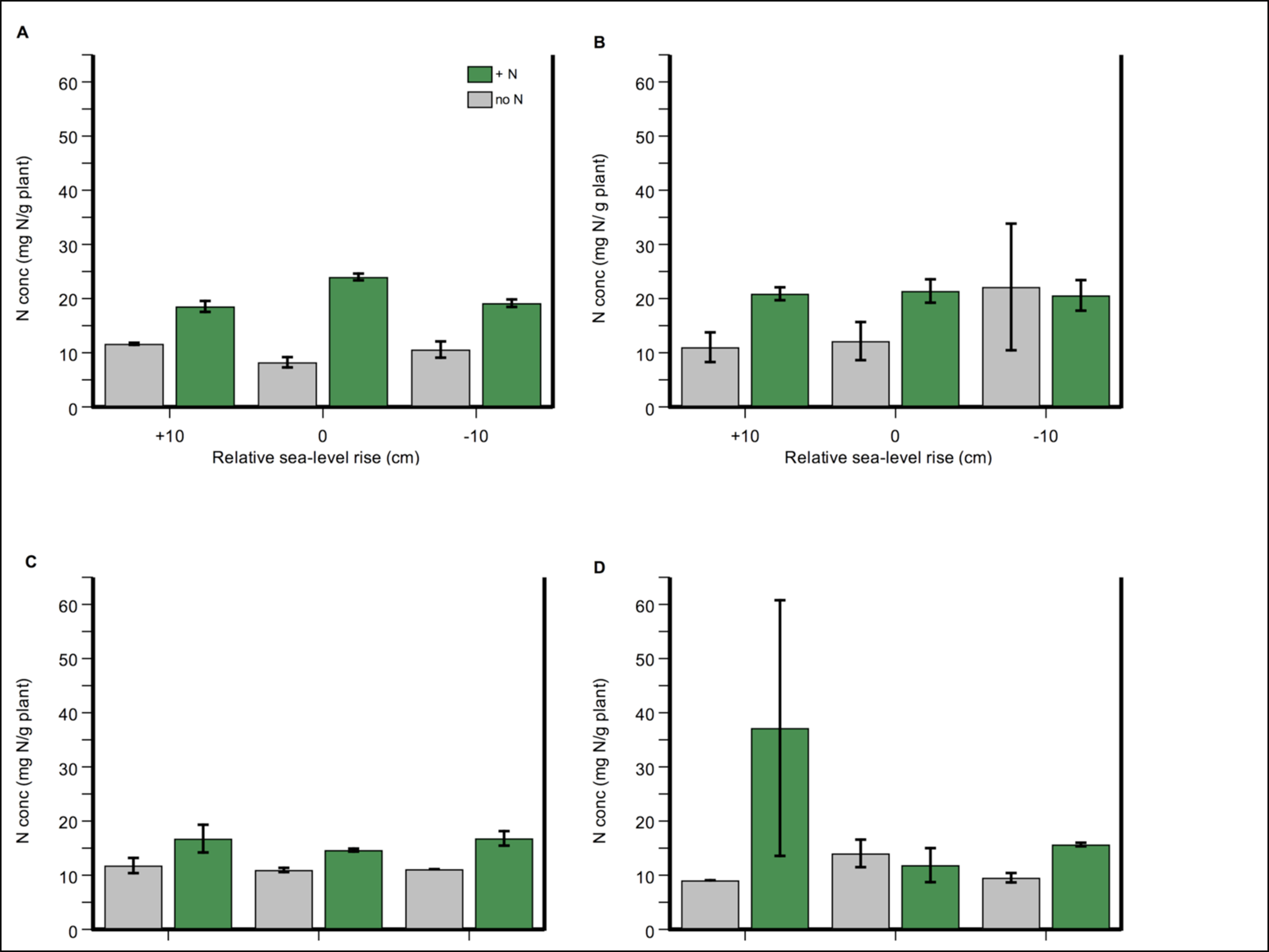

Supplement: Figure S2 — Nitrogen concentration in aboveground plant tissue increased strongly in plots with nitrogen addition. N concentration ([N]) (mgN g−1 plant tissue) in a) July 2008; b) Nov 2008; c) July 2009; and d) Nov 2009 harvests. Four out of five harvests are shown. Control treatment (no N) is shown in grey, and N-addition treatment (+N) in green. Error bars depict standard error of the mean. (TIF) [file pone.0038558.s002.tif]
